# Sustained-Paced Finger Tapping: A Novel Approach to Measure Internal Sustained Attention

**DOI:** 10.3389/fpsyg.2018.00881

**Published:** 2018-05-30

**Authors:** Marco A. Petilli, Daniela C. Trisolini, Roberta Daini

**Affiliations:** ^1^Department of Psychology, University of Milano-Bicocca, Milan, Italy; ^2^NeuroMI – Milan Center for Neuroscience, Milan, Italy; ^3^COMiB – Optics and Optometry Research Center, University of Milano-Bicocca, Milan, Italy

**Keywords:** test development, sustained attention, internal attention, assessment, S-PFT

## Abstract

Sustained attention is a fundamental prerequisite for all cognitive functions and its impairment is a common aftermath of both developmental and acquired neurological disorders. To date, all the sustained attention tasks rely heavily on selective attention to external stimuli. The interaction between selective and sustained attention represents a limit in the field of assessment and may mislead researchers or distort conclusions. The aim of the present perspective study was to propose a sustained version of the Paced Finger Tapping (S-PFT) test as a novel approach to measure sustained attention that does not leverage external stimuli. Here, we administered S-PFT and other attentional tasks (visual sustained attention, visuospatial attention capacity, selective attention, and divided attention tasks) to 85 adolescents. Thus, we provide evidence suggesting that S-PFT is effective in causing performance decrement over time, an important trademark of sustained attention tasks. We also present descriptive statistics showing the relationship between S-PFT and the other attentional tasks. These analyses show that, unlike visual sustained attention tests, performances to our task of internal sustained attention were not correlated to measures of selective attention and visuospatial attention capacity. Our results suggest that S-PFT could represent a promising and alternative tool both for empirical research and clinical assessment of sustained attention.

## Introduction

Sustained attention, or vigilance, is the ability to self-sustain the processing of stimuli whose repetitive and non-arousing qualities would otherwise lead to habituation and distraction by other stimuli (Robertson et al., 1997). The ‘vigilance level’ and the ‘vigilance decrement’ are the two indices mostly considered: the first refers to the overall ability to detect signals while the second refers to the decline over time in attention-requiring performance (Sarter et al., 2001).

In both research and clinical settings, a number of measures of sustained attention have been employed ranging from paper-and-pencil to computerized tasks (Ballard, 1996; Lezak, 2004). Typically, these tasks involve the maintenance in working memory of auditory or visual items, which could be frequent or rare in time, high or low in salience and complexity (Hubal et al., 2009). Despite the wide variety of measures of sustained attention that have been proposed, all of these tools involve the continuous selection of perceptual stimuli. In clinical settings, this represents a great constraint, considering the high frequency of neurological disorders which compromise selective attention. Moreover, it is worth noting that exogenous stimuli can activate and enhance the vigilance level (Manly et al., 2002). The interaction between stimulus-driven and endogenous attention represents a serious limit in experimental fields and may mislead researchers or distort conclusions. For instance, some studies have investigated the effect of everyday activity, such as sports practice (Zwierko et al., 2014) or video game playing (Trisolini et al., 2017) on visual sustained attention. Since it is known that sports and videogames practice improve selective attention (Green and Bavelier, 2003; Memmert, 2009), it follows that visual sustained attention tasks cannot disentangle the combined effects of selective and sustained attention in these samples. Although a comprehensive and valid evaluation of sustained attention must rely on various, and heterogeneous, measures, to date no alternative to external sustained attention tasks has been available.

Here, we propose the Sustained-Paced Finger Tapping (S-PFT) as a new approach to measure sustained attention based on the maintenance of internal mental representations (i.e., internal attention^1^) rather than exogenous stimulations. This test is a variant of the Paced Finger Tapping (PFT), a test used to assess sensorimotor synchronization (Lewis et al., 2004) and timing of movement (Wing, 2002). In this paper we describe this novel approach. Then, we evaluate the capacity of S-PFT to detect the decrement of performance over time, which represents the most important trademark of all sustained attention tasks. Finally, we report the correlations between S-PFT and other attentional measures to highlight potential relationships between S-PFT and other attentional measures.

## S-Pft: the Task

Classic PFT uses a synchronization-continuation paradigm (Wing, 2002). During the SP a series of isochronous auditory stimuli (interstimulus intervals typically ranging from 200 to 1000 ms) are presented and the subject is required to synchronize with them by pressing a key. SP is followed by an unpaced CP in which the subject is instructed to continue responding at the same rate for a further 30–50 responses. Like PFT, S-PFT required continuous input to allow the detection of rapid fluctuations in vigilance. Like classical sustained attention tasks, S-PFT was modified to be sufficiently monotonous to allow distractibility and habituation. Main adaptations consisted of the extension of the task duration and of the adoption of a longer interstimulus interval. An ad-hoc scoring procedure was designed to detect alterations associated with participant vigilance level and vigilance decrement.

### Task Procedure

**FIGURE 1:**
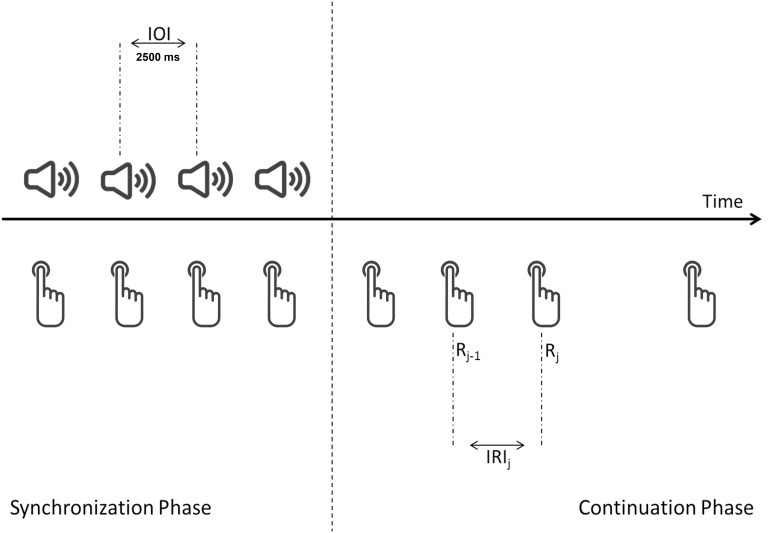
Schematic representation of the synchronization–continuation paradigm. IOI, inter-onset interval; IRI_j_, inter-responses interval; R, response.

The task was developed with the Psychophysics Toolbox (Brainard, 1997) for Matlab R2013a. During the SP, 20 isochronous auditory tones (250 Hz; 200 ms duration) was emitted at a fixed pace (interonset intervals: 2500 ms). The SP was followed by 5 min of the CP, in which no auditory stimuli were reproduced. Participants were instructed, at first, to synchronize their response with the auditory pace, and, then, to continue reproducing the same pace when the auditory tones ceased (**Figure 1**). Responses were given by pressing the keyboard spacebar with the index finger of the dominant hand.

### Scoring Procedure

**FIGURE 2:**
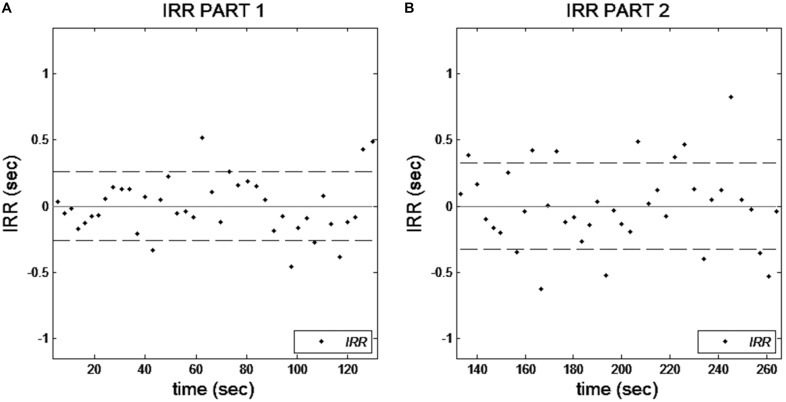
Graphical output of the task reporting the inter-response residuals (IRRs) as a function of time in the first **(A)** and the second half **(B)** of the task. The dashed lines represent the standard deviations of the IRRs. This observation allows to have an immediate clue about the presence of an increase of IRRs dispersion in Part 2 of the task compared to the Part 1.

The phase particularly relevant to our purpose was the CP, which involves the specific function to internally sustain a representation of the inter-stimulus interval. We computed, as critical responses, those tappings recorded starting from 30 s after the end of the SP. The first 30 s of CP were excluded from the analysis, since the withdrawal of external cues at the beginning of CP mainly stresses cognitive processes (memory, task-switching, or learning) (Wing, 2002) that are not relevant to our purpose. We first estimated the evolution over time of the internal representation of the pace. Then we extracted indices of vigilance based on the level of synchronization of responses with the estimated internal pace. Two main factors we expected to affect the time course of the IRI: on the one hand, potential difficulty of internally maintaining an identical representation of the pace throughout the task (i.e., memory effects) should result in smoothing and lasting changes of the IRI over time. On the other hand, the presence of lapses of attention should result in abrupt and isolated changes of the IRIs. In the light of this, to estimate the representation of the internal pace over time, the IRI × time distribution was fitted to a quadratic function, a curve that is flexible enough to fit lasting variations of the internal pace, but minimally affected by abrupt deviations in the distribution. Three indices were computed based on the IRR (i.e., the difference between each data point and the corresponding model estimate):

1. *Increase of Response Variability (SPFT-IRV)*: the standard deviation of the IRRs was computed, separately for the first (P1) and the second part (P2) of the task, to obtain measures of divergence of IRIs from the internal representation of the pace (**Figure 2**). A measure of vigilance decrement was obtained by computing the ratio between the standard deviation of IRRs in P2 and P1. Hence, the SPFT-IRV value indicated the degree to which the variability of IRIs from the internal pace representation in P2 was higher than in P1. The poorer the performance in P2 compared to P1, the higher the SPFT-IRV value.
2. *Increase of Lapses of Attention (SPFT-ILA):* Strongly deviant and positive IRRs (>3 standard deviations) have been considered as signs of attentional lapses. The number of attentional lapses in the P1 of the task was subtracted from the number of attentional lapses in the P2. Hence, the SPFT-ILA value informed about the increase of attentional lapses over time.
3. *Total Lapses of Attention (SPFT-TLA):* The sum of attentional lapses throughout the whole task was computed to provide a measure of the overall vigilance level during the task.

## S-Pft: Relationship With Other Attentional Measures

### Methods

#### Participants and Procedures

Eighty-five healthy adolescents (15–19 years old) were recruited from four high schools in Milan. Two participants were excluded because of extreme score in one task (>Q3 + 3 IQR). Participants and their parents gave informed and written consent prior to the enrolment in the study. This study was carried out with the approval of the ethical committee of University of Milano-Bicocca (Protocol No. 0000276/14 on date 08-01-2014) and in accordance with the principles expressed in the Declaration of Helsinki. Each participant completed a test battery in a session lasting around 60 min. Short breaks were given after each task.

#### Measures

In addition to the S-PFT, the battery included the following attention tests:

***Jumping Square-Sustained Attention Task (JS-SAT)*** (Zimmermann and Fimm, 2007; see Trisolini et al., 2017, for the detailed procedure). This is a 20 min, sustained visual-attention task. It requires examinees to respond as rapidly as possible when a flashing visual stimulus appears consecutively twice in the same half of a rectangle centered on the screen. Two measures of vigilance decrement and one of vigilance level were extracted:

1. *Increase of Response Time (JS-IRT):* The ratio between the standard deviation of response time (RT) of correct responses in the first and the second half of the task. Hence, the poorer the performance in P2 compared to the P1, the higher the *JS-IRT* value (i.e., a more pronounced decrement over time of vigilance).
2. *Increase of Misses (JS-IM):* The difference between the number of the missed targets in the first and the second half of the task. Hence, the poorer the performance in P2 compared to the P1, the higher the *JS-IM* value (i.e., a more pronounced increase over time of attentional lapses).
3. *Total Misses (JS-TM):* The overall number of targets missed across the whole task.

***Digit Cancelation Test (DCT)*** (see Spinnler and Tognoni, 1987 for the detailed procedure). This paper-and-pencil test requires examinees to cross out target digits in three separate and increasingly difficult visual search matrices within a set time (i.e., 45 s per matrices). The number of correct cancelations is considered an index of selective attention because it reflects the ability to focus on relevant targets while ignoring other irrelevant distractors.

***Visual Enumeration Task (VET)*** (see Green and Bavelier, 2003 for the detailed procedure). This computerized task consists of reporting the number of squares simultaneously presented on a flashing display (50 ms). Total duration of the task is around 12 min. Following the method reported by Green and Bavelier (2003), we estimated the accuracy breakpoint (i.e., the numerosity at which the performance switched from subitizing to counting), by fitting error rate data to a bilinear function with the first component of the function constrained to have a relatively flat slope (maximum slope of 3% per item), while the second was modeled as increasing linearly. The accuracy breakpoint represents the span of apprehension (i.e., number of stimuli that can be extracted from a brief exposure to a visual display), which is a measure of visuospatial attention capacity (Green and Bavelier, 2003).

***Paced Auditory Serial Addition Test (PASAT*)** (see Gronwall, 1977 for the detailed procedure). Single digit numbers are presented at a specified pace (2 s). Participants are required to add the last presented number to the one preceding it. Total duration of the task is 2 min. The final score is the number of correct responses. This task allows the evaluation of working memory, divided and selective auditory attention as well as information-processing speed.

### Results

**FIGURE 3:**
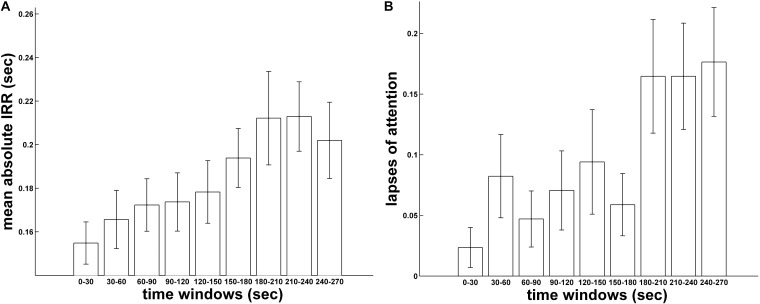
Mean absolute IRRs **(A)** and mean distribution of attentional lapses **(B)** as a function of time divided into equal size time windows of 30 s.

First, we evaluated whether and how response variability and attentional lapses changed over time. Thus, a linear trend analysis was performed both on the mean absolute IRRs and the mean number of lapses of attention over time, divided into time intervals of 30 s. As expected, both of these analyses, showed the presence of a positive linear trend (IRRs: *F*(1,84) = 23.15, *p* < 0.001, **Figure 3A**; Lapses of attention: *F*(1,84) = 17.91, *p* < 0.001, **Figure 3B**) suggestive of a clear vigilance decrement as a function of time.

To examine the relationship between the S-PFT and the JS-SAT, Pearson correlation coefficients were calculated for the three indices of S-PFT and the corresponding indices of JS-SAT. All the correlations between the variables of S-PFT and the corresponding variables of JS-SAT were statistically significant. In particular, SPFT-IRV was positively correlated to the decrement over time of RT of JS-SAT (i.e., JS-IRT) (*r* = 0.298, *p* = 0.006). SPFT-ILA was positively correlated with the increase over time of attentional lapses of JS-SAT (i.e., JS-IM) (*r* = 0.265, *p* = 0.015). Finally, SPFT-TLA was positively correlated to the overall incidence of missed target detections of JS-SAT (i.e., JS-TM) (*r* = 0.338, *p* = 0.002). Conversely, the correlations computed between the S-PFT variables and the non-equivalent variables of JS-SAT were not significant (all *p* > 0.08).

The relationship among the S-PFT, the JS-SAT and the other attentional measures were examined. No significant correlations emerged between the S-PFT indices and the other attentional measures used in this study (all *p* > 0.09). However, a significant correlation did emerge between the selective attention task (i.e., DCT) and JS-IM (*r* = 0.228, *p* = 0.04). The higher the performance in the DCT, the stronger the increase of missed target detection over time in JS-SAT. A possible explanation for this result is that individuals who showed higher selective attention in the first half of the JS-SAT may manifest more difficulty in maintaining the same attentional level over time. Therefore, these individuals may show more sensitivity to the effect of the time spent on the task. Finally, a significant correlation emerged between JS-TM and VET (*r* = -0.228, *p* = 0.045), suggesting that the lower the visuospatial attention capacity, the greater the overall incidence of attentional lapses.

To summarize correlational analysis highlighted the presence of significant correlation between the measures of S-PFT and analogs measures of JS-SAT. Moreover, they indicated that, unlike JS-SAT, in our sample, S-PFT scores were not significantly correlated with measures evaluating external components of attention, such as selective visual attention and visuospatial attention capacity.

## Conclusion

In this study, we proposed the S-PFT, a new measure of sustained attention that leverages attention to stimuli that are not present in the external environment but are internally represented and maintained. This is a 5-min task, that requires the subject to internally maintain, and repetitively reproduce, an internal representation of a pace previously memorized. In this study, S-PFT has proved to be effective in causing and evaluating the typical decrement of performances symptomatic of the decay of sustained attention over time.

Besides S-PFT, in this study other attentional tasks were administered to a sample of adolescents. Our statistical analyses suggested that S-PFT could be a promising alternative for measuring sustained attention with the advantage that, unlike typical sustained attention tasks, performances of S-PFT do not seem to be affected by stimulus-driven attentional skills (or deficits).

We believe that S-PFT could represent a useful tool to assess sustained attention both in empirical contexts and clinical settings, even with patients with exogenous attention impairment.

Additionally, by showing the relationship between a classical sustained attention task and external attention, our findings stress the importance of having tasks that leverage different cognitive domains to draw valid conclusions about sustained attention functionality. However, it is important to highlight that, as the classical sustained attention measures are affected by the presence of external attention impairment, S-PFT should not be considered a valid tool in the presence of neurological conditions affecting temporal processing, mental time-keeping or motor coordination and production. The encouraging results of this study hopefully will motivate further work to establish the psychometric properties of the S-PFT, and, at the same time, to design tools of sustained attention assessment with alternative approaches to the current ones.

## Author Contributions

All authors gave substantial contributions to the conception and the design of the work, acquisition, analysis, and interpretation of data for the work, contributed to draft the work and revise it, approved the final version of the manuscript to be published, and agreed to be accountable for all aspects of the work in ensuring that questions related to the accuracy or integrity of any part of the work are appropriately investigated and resolved.

## Conflict of Interest Statement

The authors declare that the research was conducted in the absence of any commercial or financial relationships that could be construed as a potential conflict of interest.

## Footnotes

1 With internal attention, we refer to the orienting of attention on representations that are not present in the environment but are internally generated (Zanto and Gazzaley, 2013).

## Abbreviations

CP: continuation phase
DCT: digit cancelation test
IOI: interonset interval
IRI: interresponse interval
IRR: interresponse residual
JS-IM: jumping square-sustained attention task – increase of misse
JS-IRT: jumping square-sustained attention task – increase of response time
JS-SAT: jumping square-sustained attention task
JS-TM: jumping square-sustained attention task – total misses
P1: part 1
P2: part 2
PASAT: paced auditory serial addition test
SP: synchronization phase
S-PFT: sustained-paced finger tapping
SPFT-ILA: sustained-paced finger tapping – increase of lapses of attention
SPFT-IRV: sustained-paced finger tapping – increase of response variability
SPFT-TLA: sustained-paced finger tapping – total lapses of attention
VET: visual enumeration task.
